# Evaluation of umbilical cord blood presepsin in the diagnosis of neonatal early-onset sepsis

**DOI:** 10.3389/fped.2026.1916996

**Published:** 2026-09-16

**Authors:** Huaqing Zhang, Zhihao Xiao, Yong Xia, Xiaoyan Hu

**Affiliations:** 1 Department of Pediatrics, Peking University Shenzhen Hospital, Shenzhe, Guandong, China; 2 Department of Transfusion Medicine, Peking University Shenzhen Hospital, Shenzhen, Guandong, China

**Keywords:** biomarker, early-onset sepsis, presepsin, soluble leukocyte differentiation antigen 14, umbilical cord blood

## Abstract

**Background:**

This study aimed to measure soluble leukocyte differentiation antigen 14 (presepsin) levels in umbilical cord blood from neonates with risk factors for early-onset sepsis (EOS). The objectives were to evaluate presepsin's diagnostic value for EOS and determine its optimal diagnostic cutoff in umbilical cord blood.

**Study design:**

In this single-center prospective study, we enrolled neonates born between June 2024 and September 2025 at our hospital who presented with EOS risk factors. Infants were stratified into two groups based on EOS development: the EOS group and the non-EOS group. Within 24 h of birth, umbilical cord blood and venous blood samples were collected for analysis. We measured presepsin levels in cord blood and evaluated complete blood counts, C-reactive protein (CRP), procalcitonin (PCT), and interleukin-6 (IL-6) in both cord and peripheral blood. These biomarkers were compared to assess presepsin's diagnostic performance relative to traditional inflammatory markers for neonatal EOS.

**Results:**

The study included 195 eligible neonates. Umbilical cord presepsin levels were significantly elevated in the EOS group compared to controls (1,084.51 pg/mL vs. 618.94 pg/mL, *p* < 0.001). Presepsin demonstrated higher diagnostic accuracy than traditional biomarkers (white blood cell (WBC), CRP, PCT, and IL-6) for EOS detection. Based on our analysis, the maximum Youden index was 0.726, corresponding to an optimal cutoff value of 800.91 pg/mL for umbilical cord blood presepsin in predicting neonatal EOS, with a sensitivity of 88.0%, specificity of 84.6%, and the area under the curve (AUC) of 0.941.

**Conclusion:**

Umbilical cord presepsin showed higher diagnostic accuracy than traditional inflammatory markers for neonatal EOS. and the optimal cut-off value of umbilical-blood presepsin to predict EOS was 800.91 pg/mL.

**Clinical Trial Registration**: https://www.chictr.org.cn/showproj.aspx?proj=194627, identifier ChiCTR2300072525.

## 1 Introduction

Early-onset sepsis (EOS), defined as sepsis occurring within the first 72 h of life, results from pathogenic invasion and proliferation in the neonatal bloodstream. With an incidence of 0.5–2 cases per 1,000 live births, EOS and prematurity collectively contribute to nearly 50% of global mortality in children under five years of age. The nonspecific clinical presentation and lack of consensus regarding diagnostic criteria and biomarkers make EOS identification particularly challenging for clinicians (1–3). Timely intervention is critical, as delays in antibiotic administration significantly increase mortality risk (4), underscoring the need for accurate early diagnostic tools.

Current standalone biomarkers—including complete blood counts (CBC), CRP, PCT, and IL-6—demonstrate suboptimal sensitivity and specificity for EOS detection (5). These limitations arise from physiological elevations in WBC, CRP, and PCT during the first 72 postnatal hours, coupled with IL-6's poor specificity. Furthermore, these markers are confounded by non-infectious maternal and fetal inflammatory conditions (1, 6, 7), necessitating the discovery of more reliable diagnostic alternatives.

Emerging evidence highlights presepsin as a promising biomarker for EOS diagnosis due to its resistance to perinatal confounding factors (8, 9). This N-terminal fragment of soluble CD14 subtype (sCD14-ST) is released by monocytes and macrophages upon pathogen recognition. Recent studies demonstrate that umbilical cord blood presepsin effectively predicts EOS while potentially reducing unnecessary antibiotic use (10–13). Nevertheless, research on cord blood presepsin's role in EOS remains limited. Our study aims to comprehensively evaluate its diagnostic value and establish the optimal cutoff threshold for neonatal EOS detection.

## 2 Materials & methods

### 2.1 Study design and participants

In this prospective study, we screened all neonates with EOS risk factors admitted to Peking University Shenzhen Hospital (Shenzhen, Guangdong, China) between June 2024 and September 2025. Inclusion criteria required the presence of ≥2 of the following maternal/obstetric risk factors: 1) Prolonged rupture of membranes (≥24 h), 2) Intrapartum maternal fever (>38 °C within 24 h before delivery), 3) Maternal leukocytosis (WBC ≥15 × 10^9^/L) with mother's elevated CRP (≥10 mg/L) within 24 h peri-delivery, 4) Clinical chorioamnionitis (uterine tenderness or foul-smelling amniotic fluid). Exclusion Criteria: We excluded neonates with: 1) Death within 24 h of birth or treatment withdrawal, 2) Severe congenital malformations requiring surgical intervention, 3) Lack of guardian-informed consent.

This research protocol has been reviewed and filed by the Hospital Research Ethics Committee (Ethics Approval No.: PKUSZH-Research-[2023]020; National Medical Research Registration No.: MR-44-23-033837). It has also been registered at the Chinese Clinical Trial Registry with the registration number ChiCTR2300072525, and the registration date was June 15, 2023. Written informed consent was obtained from all guardians prior to enrollment.

### 2.2 Data collection and definitions

Maternal and neonatal clinical data were systematically extracted from electronic medical records, encompassing gestational age (GA), small for gestational age (SGA) status, Apgar scores at 1 and 5 min, birth weight, infant sex, delivery mode, premature rupture of membranes (PROM), amniotic fluid characteristics (turbidity or malodor), maternal intrapartum temperature, maternal white blood cell count (WBC) during the 24 h perinatal period, and intrapartum antibiotic administration. The primary study outcome focused on assessing the predictive utility of umbilical cord Presepsin for early-onset sepsis (EOS).

Study Group Stratification: Participants were classified according to established diagnostic criteria (14): 1. EOS group: Neonates meeting full diagnostic criteria for early-onset sepsis, 2. Non-EOS group: Neonates not fulfilling EOS diagnostic criteria.

EOS diagnosis adhered to the 2024 Expert Consensus on Neonatal Sepsis Management (14), requiring both: Clinical manifestations (within 72 h postpartum): Temperature dysregulation, respiratory abnormalities, feeding difficulties, or pathological jaundice, etc (15). AND ≥1 confirmatory finding from: 1. Hematologic abnormalities (≥2 parameters): WBC ≥25 × 10^9^/L (6–72 h postnatal) or <5 × 10^9^/L (any age) or absolute neutrophil count (ANC) <1 × 10^9^/L; CRP: ≥3 mg/L (0–6 h), ≥5 mg/L (6–24 h), ≥10 mg/L (>24 h); Age-adjusted PCT elevation (considering physiological postnatal trends (16); Thrombocytopenia (<100 × 10^9^/L). 2. cerebrospinal fluid (CSF) findings consistent with purulent meningitis. 3. Molecular evidence of pathogenic DNA in blood/CSF. 4. Positive microbial cultures from blood, CSF, or sterile body fluids.

Sample-size estimation: The sample size was calculated based on the sensitivity of the diagnostic test using the formula: *N* = [(Z^2^*α*/2 × Se × (1 − Se))]/(L^2^ × P). According to reference (17), the sensitivity of presepsin for the diagnosis of sepsis was 0.875. A significance level *α* of 0.05 and an allowable error L of 0.1 were adopted. By retrospectively reviewing the clinical data of our hospital over the past one-year period, the probability of EOS among patients meeting the inclusion criteria was approximately 25%; thus, the disease prevalence P was set at 0.25. The calculated sample size *N* was 172. After accounting for a 10% loss-to-follow-up rate, the required sample size was determined to be 192 cases. A total of 195 cases were finally enrolled in this study.

All the selected research subjects had their umbilical cord blood collected at a volume of 5 mL before the placenta was delivered after the birth of the newborn, and 2 mL of venous blood was collected within 24 h after the newborn's birth for testing. The test items for the umbilical cord blood samples included: complete blood count, CRP, PCT, IL 6 and Presepsin; the venous blood samples were used for detecting complete blood count, CRP, PCT and IL 6. The collected umbilical cord blood samples were immediately placed in a 4 °C cold environment and processed with a centrifuge at 3,000 r/min for 5 min to separate the plasma. After the layering was completed, the upper layer plasma was carefully aspirated using a pipette and then aliquoted into sterile 1.5 mL cryogenic tubes. Each tube was strictly labeled with the sample number and key information and a detailed ledger was established. Finally, all the samples were quickly transferred to a −80 °C ultra-low temperature refrigerator for cryopreservation, and then frozen and thawed collectively for the detection of Presepsin. The umbilical cord blood or venous blood samples for detecting traditional inflammatory indicators were tested within 2 h after collection.

### 2.3 Analytical testing & methodology

CBCs were performed using impedance-based automated hematology analyzers. CRP concentrations were determined by immunoturbidimetric assay. For inflammatory biomarkers, procalcitonin PCT and IL-6 levels were quantified using electrochemiluminescence immunoassays. Presepsin measurements were conducted via Chemiluminescence Immunoassay (CLIA) following the manufacturer's recommended protocols.

### 2.4 Statistical analysis

Statistical analysis was performed using SPSS version 27.0 (IBM Corp, Armonk, NY). Continuous variables with normal distribution were expressed as mean ± standard deviation and compared using independent Student's *t*-tests. Non-normally distributed data were presented as median with interquartile range [M (P25-P75)] and analyzed with Mann–Whitney *U*-tests. Categorical variables were described as frequencies and percentages (%) and compared using chi-square tests.

To evaluate the diagnostic performance of presepsin for EOS, we constructed ROC curves and calculated the AUC. The optimal cutoff value was determined by maximizing the Youden index (sensitivity + specificity − 1), from which corresponding sensitivity and specificity were derived. A two-tailed *P*-value <0.05 was considered statistically significant.

## 3 Results

### 3.1 General characteristics

In this cohort study, we measured umbilical cord blood presepsin levels in 195 neonates with risk factors for early-onset sepsis (EOS). The study population comprised newborns with gestational ages ranging from 30.8 to 41.7 weeks (median 39.42 weeks), including 8 preterm (4.1%) and 187 term infants (95.9%). The delivery modes were vaginal (*n* = 145, 74.4%) and cesarean section (*n* = 50, 25.6%).

The EOS group included 50 neonates (25.6%), consisting of 2 culture-confirmed cases [Group B Streptococcus (GBS) and Pseudomonas aeruginosa] and 48 clinically diagnosed cases. The non-EOS control group contained 145 neonates (74.4%) who either showed no EOS symptoms within 72 postnatal hours or had negative blood cultures leading to antibiotic discontinuation.

Comparative analysis of demographic and perinatal characteristics (see Supplementary Table 1) revealed no significant intergroup differences (*P* > 0.05) in: Gestational age, Neonatal sex distribution, Birth weight, Delivery mode, Apgar scores (1- and 5-minute), Maternal intrapartum temperature, Maternal white blood cell counts (24 h pre-/post-delivery), Intrapartum antibiotic prophylaxis, Duration of premature rupture of membranes, Amniotic fluid characteristics (turbidity/odor).

### 3.2 Analysis of umbilical cord blood biomarkers

Comparative evaluation of cord blood biomarkers between groups revealed distinct profiles (see Supplementary Table 2): CRP, PCT, neutrophil percentage (*N*%), and platelet count (PLT) showed comparable levels between EOS and non-EOS neonates. the two groups were not statistically significant (*P* > 0.05);The study revealed two distinct tiers of biomarker performance in umbilical cord blood: 1. Marginally Significant Markers: While IL-6 (18.95 vs. 12.05 pg/mL) and WBC (16.21 vs. 14.7 × 10^9^/L) showed statistically significant elevations in the EOS group (both *P* < 0.05), their absolute values remained within relatively low clinical ranges. This suggests their limited utility as standalone diagnostic indicators for EOS. 2. Highly Discriminative Biomarker: Presepsin demonstrated striking differential expression:- EOS group: 1,084.51 pg/mL (IQR: 853.33–1,238.48), Non-EOS group: 618.94 pg/mL (IQR: 507.45–759.55); This nearly two-fold elevation (*P* < 0.001) maintained robust statistical significance while existing in clinically meaningful ranges, highlighting its potential as a superior diagnostic marker compared to conventional inflammatory parameters.

### 3.3 Venous blood indicators

This study compared the differences of various laboratory indicators in the venous blood between the two groups. The results showed (see Supplementary Table 3): In the venous blood, the median levels of CRP, PCT, IL-6, WBC, and the percentage of N% in the EOS group were 2.19 mg/L, 2.02 ng/L, 52.1 pg/mL, 21.84 × 10^9^/L, and 73% respectively, which were all higher than those in the non-EOS group, and the differences were statistically significant (*P* < 0.05).

### 3.4 Diagnostic value

This study utilized the ROC curve and based on the principle of the maximum Youden index to determine the efficacy of Presepsin in diagnosing EOS and the optimal cut-off value for Presepsin in diagnosing EOS. The results are as follows (see Supplementary Figure 1 and Supplementary Table 4): In terms of using inflammatory indicators to diagnose EOS, the levels of IL-6, WBC in cord blood, and WBC, CRP, PCT, and N% in venous blood have moderate efficacy in diagnosing EOS. Their AUC, sensitivity, and specificity are significantly lower than those of cord blood Presepsin. Cord blood Presepsin demonstrates the most outstanding diagnostic performance: when the optimal cut-off value is 800.91 pg/mL, the AUC is 0.941, the 95% confidence interval (95% CI) is 0.904–0.979, corresponding to a sensitivity of 88.0% and a specificity of 84.6%. The maximum Youden index is 0.726.Among the two confirmed cases of EOS with positive blood cultures: One case was caused by GBS with a Presepsin level of 1,268.76 pg/mL; The other case was caused by Pseudomonas aeruginosa with a Presepsin level of 1,686.84 pg/mL.

### 3.5 The impact of antibiotics

We performed an internal comparison of cord blood presepsin between the two study groups (EOS vs. non-EOS) to assess whether maternal antibiotic use influenced biomarker levels. The results showed that the presepsin levels in umbilical cord blood were not affected by the use of antibiotics before birth, with *P* > 0.05 (see Supplementary Table 5). This finding supports presepsin's stability as an EOS diagnostic marker, independent of prenatal antibiotic administration.

## 4 Discussion

This study measured umbilical cord blood Presepsin levels in neonates with risk factors for EOS and compared them with traditional inflammatory markers. It confirmed a significant correlation between umbilical cord blood Presepsin and EOS, demonstrated that Presepsin outperforms traditional inflammatory markers in diagnosing EOS, and established a cutoff value for Presepsin.

Neonatal sepsis is one of the most common causes of mortality in the pediatric population. It is defined as an infection occurring in the neonatal period where pathogens invade the bloodstream, multiply, and cause systemic illness. Due to the subtle and nonspecific early signs and symptoms of EOS, delayed diagnosis can lead to disease progression and increased mortality. Thus, early recognition and diagnosis of EOS are crucial.

The diagnostic gold standard for EOS is blood culture, but the positivity rate of blood culture is low. The positivity rate of blood culture in high-risk EOS neonates is less than 1%. There are also literature reports that the blood culture positivity rate in EOS children is not very high either. The blood culture positivity rate of EOS children in developed countries ranges from 1:17 to 1:7, and the global blood culture positivity rate of EOS children is between 6.7% and 55.4% (18). The use of prophylactic antibiotics by mothers before delivery and the small blood volume (only 1 mm) collected from newborns, will further reduce the positivity rate of blood culture (19, 20). Moreover, blood culture results are slow, usually requiring at least 48–72 h of culture, which will greatly delay the treatment time for septicemic infants. In our study, the blood culture positivity rate of EOS children was 4%, slightly lower than the global range, which may be related to the use of prophylactic antibiotics by the mothers before delivery and the small blood volume collected from the children (only 1 mL).

Consequently, all neonates with EOS risk factors or clinical suspicion of EOS are empirically treated with antibiotics, leading to unnecessary antibiotic exposure in non-infected infants. This overuse can contribute to adverse outcomes such as necrotizing enterocolitis, gut microbiota dysbiosis, and antibiotic resistance (21). Therefore, identifying high-risk neonates early using biomarkers could ensure timely antibiotic administration for true EOS cases while reducing unnecessary antibiotic use, thereby improving neonatal antibiotic stewardship.

To date, multiple biomarkers have been used to assess EOS, but relying on a single traditional inflammatory marker (such as CRP, PCT, or CBC) for early infection detection has proven inadequate (22). Combining multiple markers increases blood sampling volume, posing a risk of iatrogenic anemia in neonates. Given the low sensitivity and delayed results of blood cultures, there is an urgent need for a novel biomarker to facilitate early EOS detection. Previous studies support the use of Presepsin for EOS diagnosis, showing comparable or superior diagnostic accuracy compared to other inflammatory markers (9, 10, 22–24). A recent meta-analysis (22) reported a pooled sensitivity of 82% (95% CI: 0.62–0.93) and specificity of 86% (95% CI: 0.73–0.93) for Presepsin in diagnosing EOS. However, no definitive cutoff value for Presepsin in EOS diagnosis has been established.

Presepsin is immediately released from macrophages at the onset of infection and rapidly increases, peaking within 3 h, and perinatal factors have little impact on its levels (8, 25). In theory, it is a biomarker with high specificity for identifying bacterial infections. It can be measured in umbilical cord blood, neonatal urine, or saliva, enabling noninvasive detection (11, 26). Incorporating Presepsin into clinical practice may reduce false-positive sepsis diagnoses, unnecessary antibiotic use, and invasive procedures.

Priolo et al.'s (17) prospective clinical study found that Presepsin was significantly correlated with EOS. The Presepsin values in EOS neonates were significantly higher than those in non-EOS neonates (909 pg/mL vs. 467 pg/mL, *P* < 0.05), with the optimal cut-off value being 579 pg/mL, corresponding to a sensitivity and specificity of 87.5% and 71.8%, respectively. The use of antibiotics in pregnant women before delivery did not affect the Presepsin values in cord blood. Our study has similarities with their results.

Our study included 195 neonates, among whom there were 50 cases in the EOS group. In each group, the analysis of the use of antibiotics by mothers before delivery did not affect the results of Presepsin. Within the EOS group, the comparison of Presepsin values between those who used antibiotics before delivery and those who did not was (1,087.8 pg/mL vs. 1,056.08 pg/mL), and in the non-EOS group, the comparison was (666.84 pg/mL vs. 607.14 pg/mL), with *P* > 0.05, indicating no statistically significant differences. This is consistent with the results of Priolo (17). We also found a significant correlation between Presepsin and EOS. However, Priolo (17) included fewer cases (93), with 31.2% being preterm infants, while our study included more cases, focusing mainly on full-term populations. The Presepsin values we obtained were higher, with significant differences between the EOS group and non-EOS neonates (1,084.51 pg/mL vs. 620.07 pg/mL), *P* < 0.05, with statistically significant differences. In our cohort, the maximum Youden index was 0.726, corresponding to an optimal cutoff value of 800.91 pg/mL for umbilical cord blood presepsin in predicting neonatal EOS, with a sensitivity of 88.0%, specificity of 84.6%, and the area under the curve (AUC) of 0.941. The optimal cut-off values of Presepsin were higher than those in previous studies (12, 13, 17, 27).

In previous studies, Nusman et al. (12) aimed to verify whether a Presepsin cut-off value of 645 pg/mL could reduce the use of antibiotics in preterm infants, rather than measuring Presepsin values. Dierikx et al. (13)'s study included both full-term and preterm infants but did not compare Presepsin with traditional indicators. Aggarwal et al. (27)'s study included both preterm and full-term infants, but did not measure CBC, nor did it detect the differences between Presepsin and interleukin 6 and Fetuin, with unclear descriptions of cut-off values.

We further compared umbilical cord blood Presepsin with traditional inflammatory markers (CRP, PCT, IL-6, CBC) in both umbilical and venous blood. Among umbilical cord blood markers, IL-6, WBC, and Presepsin showed statistically significant diagnostic value for EOS, but IL-6 and WBC levels were low (median: 18.95 pg/mL and 16.21 × 10^9^/L, respectively), limiting clinical utility. In venous blood, all markers except platelets (WBC, N%, PCT, CRP, IL-6) were significantly associated with EOS. However, when comparing the diagnostic performance of umbilical cord blood Presepsin alone vs. venous blood markers, Presepsin exhibited the highest AUC (0.941), Youden index (0.726), sensitivity (0.88), and specificity (0.846) at the 800.91 pg/mL cutoff, Presepsin showed higher diagnostic accuracy than traditional inflammatory markers. This diagnostic cutoff value represents an exploratory finding from this study and requires further validation in independent cohorts.

The diagnostic criteria for EOS adopted in this study are derived from a recently-formulated Chinese expert consensus, which is widely used by neonatologists in China. Although blood culture is the gold standard for EOS diagnosis, its positive rate is extremely low. Accordingly, other non-specific biomarkers (e.g., WBC, CRP, PCT) are incorporated into the diagnostic criteria to assist clinical diagnosis. This may lead to an overestimation of the diagnostic performance of WBC, CRP and PCT, whereas the diagnostic performance of presepsin remains unaffected. Nevertheless, the results of the present study still demonstrate that presepsin has higher diagnostic accuracy than traditional inflammatory markers, further indicating that presepsin represents an ideal biomarker for EOS diagnosis.

This study has several limitations. First, the number of newborns included is limited, and the potential quantitative heterogeneity between the studied subgroups may affect the precision of statistical analysis. Second, due to the lack of positive blood culture samples in the cohort, it is not possible to evaluate the group of newborns diagnosed with sepsis, which may affect the determination of the Presepsin threshold. Third, our results lack the venous blood Presepsin concentrations. Based on our data, we suggest that future studies should further investigate whether there is a difference in the venous and cord blood Presepsin in these populations for the diagnosis of EOS.

## 5 Conclusion

Our study suggests that Umbilical cord blood Presepsin is a useful biomarker for early EOS detection. Umbilical cord blood Presepsin alone has higher diagnostic accuracy than traditional inflammatory markers (WBC, N%, CRP, PCT, and IL-6) in predicting sepsis. The optimal cutoff was 800.91 pg/mL (sensitivity: 88.0%, specificity: 84.6%). These findings provide a reference for clinical applications of umbilical cord blood Presepsin in EOS prediction.

## Data Availability

The datasets presented in this study can be found in online repositories. The names of the repository/repositories and accession number(s) can be found in the article/Supplementary Material.
