# Supplementary material for: Evaluation of umbilical cord blood presepsin in the diagnosis of neonatal early-onset sepsis
Source: Front Pediatr. 2026 Sep 16;14:1916996. doi: 10.3389/fped.2026.1916996 (PMC13623839; doi:10.3389/fped.2026.1916996)
Supplement: Supplementary file 1 [file Supplementaryfile1.docx]

| Variables | no EOS group（n=145） | EOS group（n=50） | statistic | P |
| --- | --- | --- | --- | --- |
| PROM≥24h,n(%) | 31（21.4%） | 11（22.0%） | χ2=0.008 | 0.927 |
| maternal antepartum temperature≥38.0℃,n(%) | 57（39.3%） | 26（52%） | χ2=2.499 | 0.118 |
| maternal leucocytosis≥15*10^9^/L,n(%) | 43（29.7%） | 18（36%） | χ2=0.696 | 0.404 |
| intrapartum antibiotic prophylaxis,n(%) | 74（51.0%） | 26（52.0%） | χ2=0.014 | 0.906 |
| turbid or smelly amniotic fluid,n(%) | 36（24.8%） | 14（28%） | χ2=0.196 | 0.658 |
| SGA,n(%) | 17（11.7%） | 4（8.0%） | χ2=0.537 | 0.464 |
| vaginal delivery,n(%) | 108（74.5%） | 37（74%） | χ2=0.005 | 0.494 |
| GA, weeks,M (Q₁, Q₃) | 39.71  (39.00, 40.29) | 39.36  (38.71, 39.75) | χ2=3.618# | 0.059 |
| BW, grams,M (Q₁, Q₃) | 3200  (2950,3450) | 3200  (3000,3450) | χ2=-0.02# | 0.984 |
| Sex,Males,n(%) | 76（52.4%） | 31（62%） | χ2=1.38 | 0.24 |
| 1-min Apgar score,M(Q₁, Q₃) | 9（9，9） | 9（9，9） | χ2=-0.204# | 0.839 |
| 5-min Apgar score,M(Q₁, Q₃) | 10（10，10） | 10（10，10） | χ2=-0.791# | 0.429 |

Table 1 Analysis of Perinatal and Neonatal Data of the Two Groups of Parturients

#,Wilcoxon Rank-Sum Test;χ2, chi-square test; M, median; Q₁, 1st quartile; Q₃, 3st quartile; PPROM, premature rupture of membranes; GA, gestational age; BW, birth weight; SGA, small for gestational age;EOS,early onset sepsis;* P < 0.05

Table 2 Comparison of various inflammatory markers in umbilical cord blood between the two groups

| inflammatory indicators | EOS group(n=50) | no EOS group(n=145) | Z | P |
| --- | --- | --- | --- | --- |
| Umbilical cord Presepsin（pg/ml） | 1084.51  （853.33,1238.48） | 620.07  （508.82,760.69） | -9.21 | ＜0.001* |
| Umbilical cord CRP（mg/L） | 0.2  (0.2,0.2) | 0.2  （0.2,0.2） | -0.496 | 0.62 |
| Umbilical cord PCT(ng/L) | 0.13  （0.1,0.18） | 0.12  （0.09,0.15） | -1.699 | 0.089 |
| Umbilical cord IL-6 (pg/ml) | 18.95  （7.82，80.20） | 12.05  （5.89,25.13） | -2.206 | 0.027* |
| Umbil cal cord WBC (*10^9^/L) | 16.21  （13.57，18.87） | 14.7  （11.91,17.53） | -2.143 | 0.032* |
| Umbilical cord N%(%) | 61.2  （55.15，65.58） | 61.55  （56.53,67.55） | -0.501 | 0.616 |
| Umbilical cord PLT(*10^9^/L) | 304.5  （244.00，356.25） | 302  （255.25,335.00） | -0.371 | 0.71 |

CRP, C-reactive protein; PCT,procalcitonin; IL-6,interleukin-6; WBC,white blood cells; N%,the percentage of neutrophils; PLT,blood platelet; EOS,early onset sepsis;* P < 0.05

Table 3 Comparison of various inflammatory indicators in venous blood between the two groups

| inflammatory indicators | no EOS group（n=145） | EOS group  (n=50) | Z | P |
| --- | --- | --- | --- | --- |
| venous blood CRP（mg/L） | 0.41  （0.2,1.34） | 2.19  （0.62,6.23） | -5.696 | 0* |
| venous blood PCT（ng/L） | 0.56  （0.24,1.17） | 2.02  （0.76,5.88） | -5.526 | 0* |
| venous blood IL-6（pg/ml） | 28.25  （14.8,70.88） | 52.1  （30.2,375） | -4.237 | 0* |
| venous blood WBC（*109/L） | 17.98  （13.45,22.4） | 21.84  （18.16,26.23） | -3.356 | 0.001* |
| venous bloodN% | 68.95  （64.63,74.88） | 73  （67.8,77.6） | -2.621 | 0.009* |
| venous blood PLT（*109/L） | 279.5  (245,313.25) | 271  (234,318) | -0.845 | 0.398 |

CRP, C-reactive protein; PCT,procalcitonin; IL-6,interleukin-6; WBC,white blood cells; N%,the percentage of neutrophils; PLT,blood platelet; EOS,early onset sepsis;* P < 0.05

Table 4 Analysis of the Value of Each Indicator in Diagnosing EOS

| inflammatory indicators | AUC | 95%CI | Optimal cutoff value | sensitivity | specificity | maximum Youden index |
| --- | --- | --- | --- | --- | --- | --- |
| Umbilical cordPresepsin | 0.941 | 0.904，0.979 | 800.91pg/ml | 0.88 | 0.846 | 0.726 |
| Umbilical cordIL-6 | 0.591 | 0.494,0.687 | 14.65pg/ml | 0.66 | 0.569 | 0.229 |
| Umbilical cordWBC | 0.587 | 0.497,0.678 | 17.18*109/L | 0.46 | 0.715 | 0.175 |
| Umbilical cordCRP | 0.77 | 0.687,0.853 | 1.87mg/L | 0.58 | 0.87 | 0.45 |
| venous bloodPCT | 0.77 | 0.688,0.852 | 1.37ng/L | 0.64 | 0.821 | 0.461 |
| venous bloodWBC | 0.664 | 0.577,0.751 | 21.43＊109/L | 0.58 | 0.691 | 0.271 |
| venous bloodN% | 0.624 | 0.537,0.712 | 69.55% | 0.74 | 0.488 | 0.228 |

Table 5 The effect of prenatal use of antibiotics on Presepsin in the two groups

| group | Prenatal antibiotics | no Prenatal antibiotics | Z | P |
| --- | --- | --- | --- | --- |
| EOS group | 1087.83pg/ml  (876.19, 1283.29) | 1056.08 pg/ml  (851.46,1238.48) | -0.505 | 0.614 |
| no EOS group | 666.84pg/ml  （487.35,804.29） | 607.14pg/ml  （537.43,724.59） | -0.949 | 0.342 |

EOS,early onset sepsis;* P < 0.05

Figure 1 ROC curves for each indicator in diagnosing EOS
