# Supplementary material for: Evaluation of umbilical cord blood presepsin in the diagnosis of neonatal early-onset sepsis
Source: Front Pediatr. 2026 Sep 16;14:1916996. doi: 10.3389/fped.2026.1916996 (PMC13623839; doi:10.3389/fped.2026.1916996)
Supplement: Supplementary file 2 [file Supplementaryfile2.docx]

Supplementary Table 6. Definitions, data sources, and timing of assessment of study variables

| **Variable** | **Definition** | **Data source** | **Timing of assessment** |
| --- | --- | --- | --- |
| Gestational age (GA) | Best obstetric estimate based on last menstrual period and/or early ultrasound | Electronic medical record (EMR), obstetric record | At birth |
| Birth weight (BW) | Measured within 1 hour after birth using calibrated scale | Neonatal medical record | At birth |
| premature rupture of membranes (PROM) | Rupture of membranes occurring 24 hours before delivery | Obstetric record | Before delivery |
| Intrapartum antibiotic prophylaxis | Whether antibiotics were administered to the mother within 24 hours before delivery | Obstetric medication record | Prenatal period |
| maternal leucocytosis | Maternal white blood cell count within 24 hours before delivery | Obstetric record | Before delivery |
| maternal antepartum temperature | Highest maternal body temperature within 24 hours before delivery | Obstetric record | Before delivery |
| Amniotic fluid characteristics | turbidity/odor | Obstetric record | At birth |
| Mode of delivery | Vaginal delivery or cesarean section | Delivery record | At birth |
| 1-minute Apgar score (AP1) | Standard Apgar score assessed at 1 minute after birth | Delivery record | 1 minute after birth |
| 5-minute Apgar score (AP5) | Standard Apgar score assessed at 5 minutes after birth | Delivery record | 5 minutes after birth |
| Sex | Male or female | Neonatal record | At birth |
| Birth weight to gestational age ratio (RBG) | Classified as AGA, SGA, or LGA based on population-specific growth standards | Neonatal record | At birth |
| Early-onset sepsis (EOS) | Infection occurring within 72 hours after birth, diagnosed clinically and/or confirmed by sterile body fluid culture | NICU record | ≤72 hours after birth |
